# Reviewed article Silva GA, Santos CH, Leão-Cordeiro JAB, Silva AMTC. Temporal trends and regional disparities in mortality from acute myocardial infarction in Brazil: a time series study, 2015-2024. Epidemiol Serv Saude. 2026:35;e20260533

**DOI:** 10.1590/S2237-96222026v35e20260533.a

**Published:** 2026-09-25

**Authors:** Dalila de Carvalho Silva Gonzaga

**Affiliations:** 1 Universidade de Brasília, Brasília, DF, Brazil Universidade de Brasília Brasília DF Brazil

## Peer review A

## Questionnaire

Does the manuscript contain new and significant information to justify publication? Yes

Does the Abstract (Summary) clearly and accurately describe the content of the article? Yes

Is the problem significant and concisely stated? Yes

Are the methods described comprehensively? Yes

Are the interpretations and conclusions justified by the results? Yes

Is adequate reference made to other work in the field? No

## Manuscript Structure

Length of article is: Adequate

Number of tables is: Adequate

Number of figures is: Adequate

Please state any conflict(s) of interest that you have in relation to the review of this paper. None

Interest: Good

Quality: Good

Originality: Average

Overall: Good

Recommendation: Minor Revision

## Comments

Prezados autores,

O artigo está bom, porém necessita de alguns ajustes para ser aceito.

Segue alguns ajustes para o manuscrito RESS-2026-0533:

1. Sugestão de título: O título do artigo está de acordo com as regras da RESS. Porém, segue a sugestão de título: Tendências temporais e disparidades regionais da mortalidade por Infarto Agudo do Miocárdio no Brasil, 2015-2024”

Título: informar o tema principal, delineamento, local e ano (s) da pesquisa, em consonância com o guia de redação aplicável. Títulos devem ser diretos, objetivos e sem siglas.

2. Resumo: retirar a seguinte frase da Conclusão do Resumo: Mortalidade por infarto agudo do miocárdio no Brasil. Essa frase já se repete no início do resumo. Melhorar a conclusão do resumo.

3. Palavras-chaves: Substituir as palavras-chaves que já estão no título do artigo.

Exemplos: infarto do miocárdio, mortalidade, O ideal é ter palavras-chaves que não se repete no título. Segue o link para buscar novas palavras: https://decs.bvsalud.org/

4. Aspectos éticos: usar a frase padrão da RESS: Esta pesquisa utilizou bancos de dados de domínio público e anonimizados.

5. Introdução: A introdução está bem escrita. Porém, necessita de alguns ajustes. A palavra Brasil se repete 19 vezes ao longo do texto, do resumo até a discussão. Por favor, tente reduzir. Escrever covid-19 sempre com letras minúsculas, porque é padrão da revista. O segundo parágrafo da introdução está confuso, por gentileza, reescrevê-lo de forma mais clara e direta. Se a frase ocupar muitas linhas, revise-a e busque maneiras de torná-la mais concisa: dívida em frases menores ou elimine palavras desnecessárias. Na secção Populações e estratificações: retirar todas as palavras entre parênteses e colocá-las entre vírgulas. Exemplos: (Norte, Nordeste, Sudeste, Sul e Centro-Oeste). O padrão da revista é raça/cor da pele. Por favor, substituir raça/cor por raça/cor da pele em todo o texto.

6. Métodos: Retira os Aspectos Éticos dos Métodos. Não é necessário escrever novamente nos métodos.

7. Resultados: O padrão da revista é raça/cor da pele. Por favor, substituir raça/cor por raça/cor da pele nos resultados.

8. Discussão: A frase: “A mortalidade por infarto agudo do miocárdio no Brasil”se repete muito ao longo de todo o texto. Por favor, reescrever a frase de outra forma. Seria interessante colocar as limitações do estudo nos últimos parágrafos, antes da conclusão.

No último parágrafo da discussão é importante reforçar a importância de políticas públicas e as limitações do estudo. Escrever covid-19 com letras minúsculas.

9. Figuras 1, 2, 3 e 4: Certifique-se de que todas as imagens, gráficos, figuras e mapas sejam nítidos, legíveis, inclusive seu texto, tenham escalas compatíveis e sejam de alta qualidade, com legibilidade e tamanho de fonte adequados para publicação.

10. O artigo possui poucas referências. A RESS permite até 30 referências. Para enriquecer o estudo, por gentileza, buscar mais referências.

11. Referências bibliográficas: Revisar todas as referências. O formato para citar artigos científicos segue o padrão:

Autor(es). Título. Nome abreviado do periódico. Ano;Volume(Número):Página. (com a página final abreviada [ex.: 123-5] ou página eletrônica [e-page]). Link do DOI. Abaixo seguem exemplos de tipos de documentos na norma.

Artigo científico

Com número de página inicial e final

Van der Geer J, Hanraads JAJ, Lupton RA. The art of writing a scientific article. J Sci Commun 2020;163:51-9. https://doi.org/10.1016/j.sc.2020.00372.

Com número de artigo (epage). Exemplo: Van der Geer J, Handgraaf T, Lupton RA. The art of writing a scientific article. Heliyon. 2022;19:e00205. https://doi.org/10.1016/j.heliyon.2022.e00205

